# Musculoskeletal "don't touch" lesions: pictorial essay

**DOI:** 10.1590/0100-3984.2016.0225

**Published:** 2019

**Authors:** Eduardo Kaiser Ururahy Nunes Fonseca, Adham do Amaral e Castro, Rafael Seiji Kubo, Frederico Celestino Miranda, Atul Kumar Taneja, Durval do Carmo Barros Santos, Laércio Alberto Rosemberg

**Affiliations:** 1 Hospital Israelita Albert Einstein - Departamento de Imagem, São Paulo, SP, Brazil.

**Keywords:** Bone diseases, Muscular diseases, Diagnostic imaging, Myositis ossificans, Bone neoplasms, Doenças ósseas, Doenças musculares, Diagnóstico por imagem, Miosite ossificante, Neoplasias ósseas

## Abstract

Focal bone lesions are not uncommon findings in the daily practice of radiology. Therefore, it is essential to differentiate between lesions with aggressive, malignant potential that require action and those that have no clinical significance, many of which are variants or benign lesions, sometimes self-limited and related to reactive processes. In some cases, a diagnostic error can have catastrophic results. For example, a biopsy performed in a patient with myositis ossificans can lead to an incorrect diagnosis of sarcomatous lesions and consequently to mutilating surgical procedures. The present study reviews the main radiological aspects of the lesions that are most commonly seen in daily practice and have the potential to be confused with aggressive, malignant bone processes. We also illustrate these entities by presenting cases seen at our institution.

## Abstract (pt)

O achado de lesões ósseas focais não é incomum no dia-a-dia do radiologista. É, portanto, imprescindível saber discernir as lesões com potencial maligno agressivo, que requerem ação, das desprovidas de significado clínico, muitas destas sendo variantes da normalidade ou processos reativos benignos, às vezes, autolimitados. Em alguns casos, a confusão diagnóstica pode ter resultados catastróficos, como a realização de biópsia em casos de miosite ossificante, que pode levar ao diagnóstico incorreto de lesões de origem sarcomatosa e a cirurgias mutilantes. O presente estudo faz uma revisão dos principais aspectos radiológicos das lesões que mais comumente são vistas no dia-a-dia e que possuem potencial para causar confusão com processos ósseos malignos e agressivos. Ilustramos, ainda, essas lesões, apresentando casos do nosso serviço.

## INTRODUCTION

Focal lesions are common incidental findings in the practice of radiology. Therefore, it is essential that the radiologist be able to differentiate lesions with malignant or aggressive potential (i.e., those that need specific management) from those that pose no immediate risk. In his classic text, Helms referred to the latter group of lesions as "don't touch" bone lesions^(^1^-^5^)^. Such bone lesions, now more commonly referred to as "don't touch" lesions, are defined by characteristic imaging features, the identification of which precludes the need for additional diagnostic tests or biopsies, thereby avoiding unnecessary interventions.

This study seeks to use illustrations to review the main "don't touch" lesions. The radiologist should have general knowledge of this lesion group in order to avoid unnecessary invasive procedures that can be harmful to patients.

## TRUE LESIONS THAT ARE OBVIOUSLY BENIGN

### Cortical desmoids

**Figure 1:**
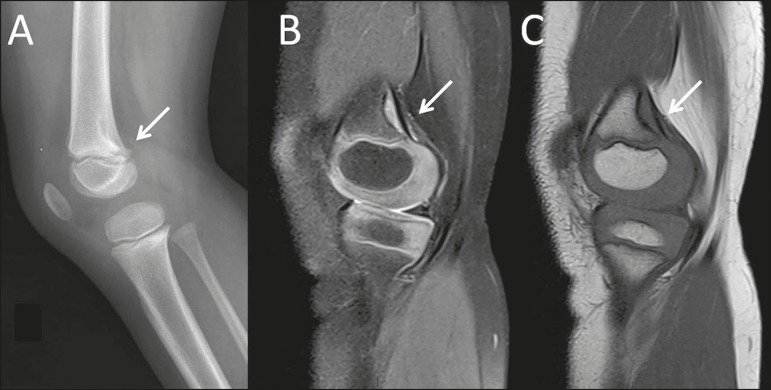
A 4-year-old female with spontaneous knee pain. Note, as an incidental finding on an X-ray of the left knee (**A**), as well as in T2-weighted and T1-weighted MRI sequences of the same knee (**B** and **C**, respectively), a typical cortical desmoid (arrows) in the distal femoral metaphysis, located in the posterior cortex.

A cortical desmoid is a benign lesion, composed of reactive fibrous tissue, that is most common in adolescents. Cortical desmoids tend to regress spontaneously. Their typical location is the posteromedial aspect of the distal femoral metaphysis, and they occur bilaterally in up to a third of cases. On a conventional X-ray, a cortical desmoid appears as an area of irregularity/cortical erosion with a sclerotic base. In this typical location and in the appropriate clinical context (Figure 1), these findings are diagnostic and no additional procedures are necessary. A biopsy is contraindicated, because of the potential for confusion with a malignant neoplasm^(^1^,^^2^^,^^6^^)^.

### Subchondral cysts

**Figure 2:**
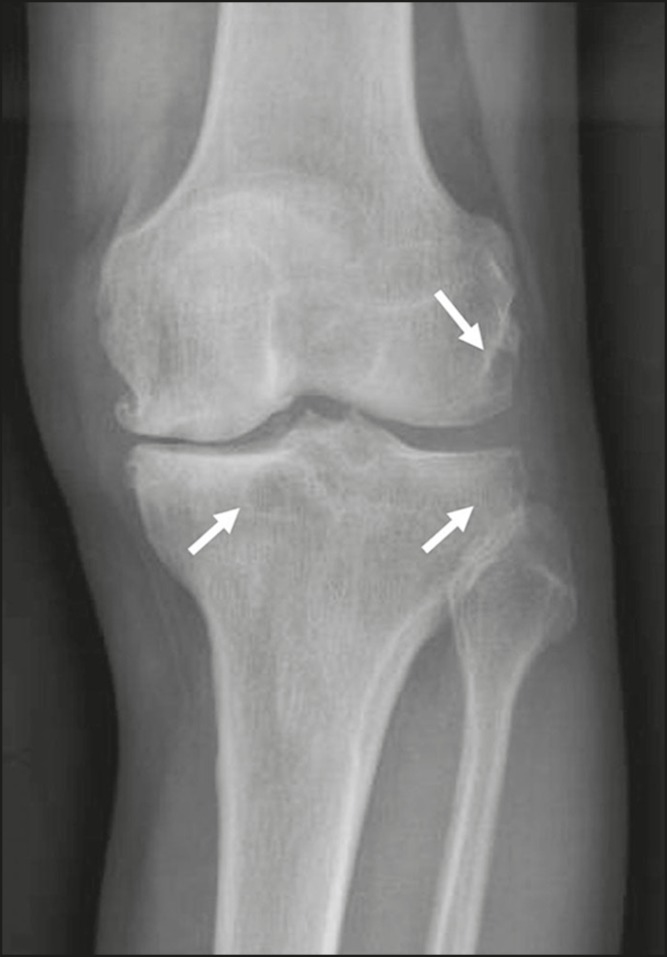
A 73-year-old male with knee pain. Anteroposterior X-ray of the left knee showing arthrosis, with subchondral cysts (arrows).

Subchondral cysts, also known as geodes, are classic alterations of osteoarthritis. The typical presentation on a simple X-ray is that of round, radiolucent lesions with well-defined borders that can appear sclerotic on computed tomography (CT). During their formation, they can present with bone edema on magnetic resonance imaging (MRI), with a small halo of enhancement after administration of gadolinium. In rare cases, there is central enhancement, denoting the presence of synovial tissue within the cyst. When large, they can mimic lytic lesions of the epiphysis. As illustrated in Figure 2, the key to making the differential diagnosis is within the context of degenerative changes that are part of the osteoarthritis spectrum^(^2^,^^6^^)^.

### Costochondritis (Tietze's syndrome)

**Figure 3:**
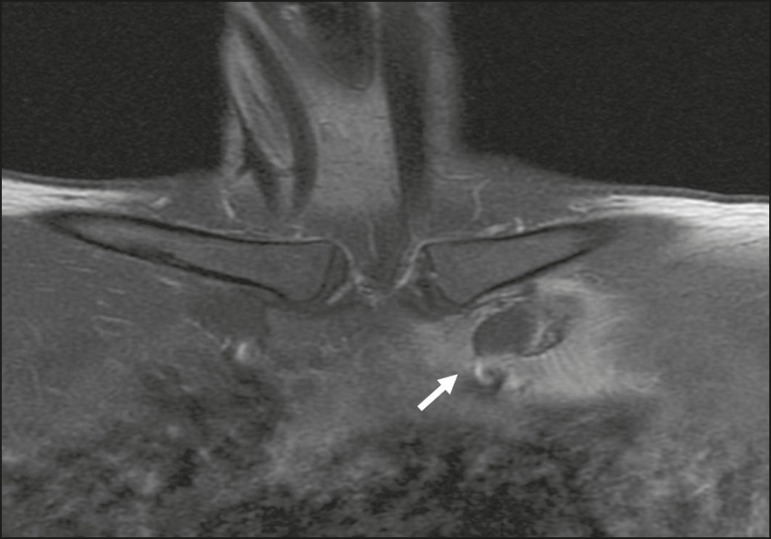
A 38-year-old female with chest wall pain. An MRI scan shows edema and enhancement of the periarticular bone (arrow) in the first left costochondral junction (in the sternal notch and in the first rib), extending to the adjacent soft tissue between the left sternoclavicular joint and the second left costochondral junction, suggesting local inflammatory changes (costochondritis), with no signs of associated fractures or collections.

Costochondritis is an inflammatory condition that presents as chondral hypertrophy of the costosternal interface, with an increase in the costal margin, and is often painful. It most often affects a single intercostal space. MRI shows thickening of the cartilages at the painful point indicated by the patient. T2-weighted MRI sequences show high signal intensity and subchondral bone edema (Figure 3). Costochondritis can also present with intense enhancement by paramagnetic contrast medium^(^10^)^.

### Small bone islands

**Figure 4:**
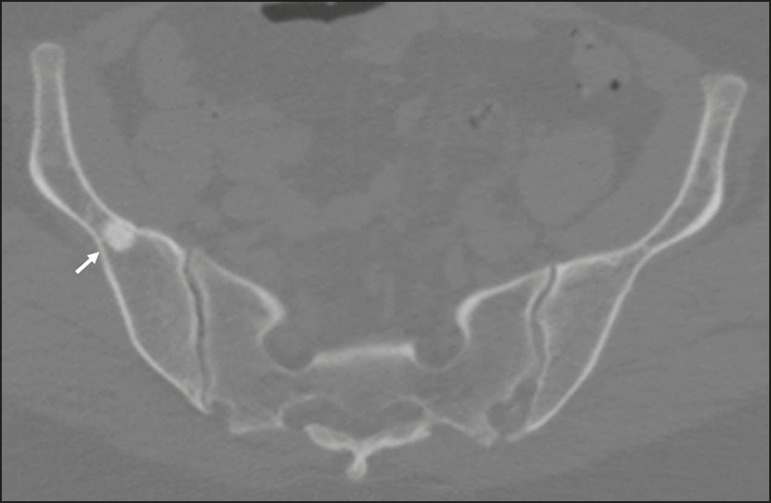
A 61-year-old female with a 10-day history of constant pain, unrelated to trauma. CT showing a small bone island (arrow) in the transition between the body and the wing of the right iliac bone.

Small bone islands (enostoses) are common incidental findings on imaging examinations. They are seen in various age groups and at various body sites, most often occurring in the pelvis, femur, or axial skeleton. They are sclerotic foci that extend to the bone trabeculae, which gives them spiculated margins (Figure 4). Due to their high calcium content, they present marked hypointense signals in all MRI sequences. Although small bone islands should not be confused with malignant sclerotic lesions, they merit invasive investigation if they show accelerated growth, defined as a ≥ 50% increase in their diameter within one year^(^1^,^^2^^,^^6^^)^. A related condition is osteopoikilosis, a hereditary disorder in which small bone islands appear in groups around several joints, predominantly affecting the long bones, tarsal bones, or carpal bones^(^1^,^^2^^,^^6^^)^.

### Fibrous dysplasia

**Figure 5:**
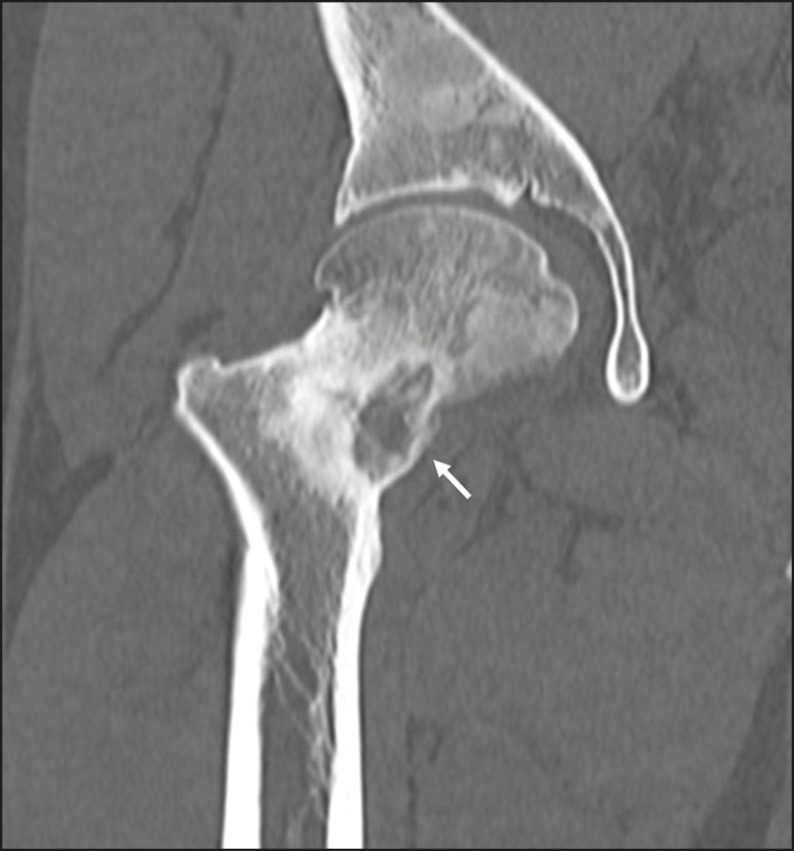
A 28-year-old asymptomatic female reporting disproportionality between the size of the bones on the right side of her body and that of those on the left side (the former being larger than the latter), with an incidental finding of fibrous dysplasia on CT. Note the areas of ground-glass attenuation (arrow).

Fibrous dysplasia is a change in bone development characterized by fibrous matrix and bone tissue, typically located in the bone marrow of the metaphyseal region in children and young adults. Its appearance on imaging examinations depends on the ratio between the fibrous matrix and the bone tissue, the typical description being that of a ground-glass pattern (Figure 5), which results from the loss of the usual bone trabeculation. Due to the loss of normal bone architecture, there is an increased risk of fractures^(^1^,^^2^^,^^6^^)^.

### Non-ossifying fibroma

**Figure 6:**
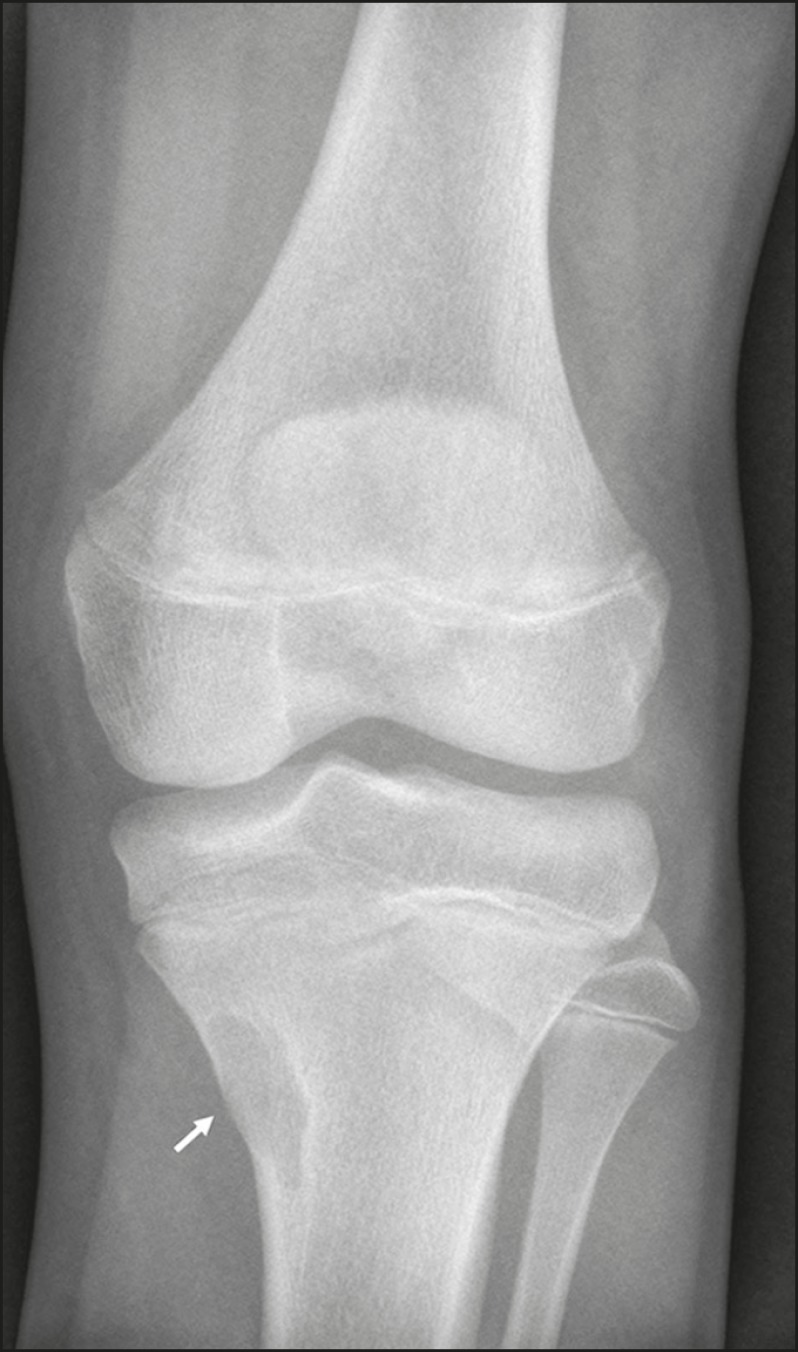
A 12-year-old male with a one-week history of pain in the medial region of the left knee. He reported no history of trauma, although he did report being a runner and playing soccer on a regular basis. A well-defined, lobulated bone lesion (arrow) with sclerotic margins in the posteromedial metadiaphyseal subcortical bone of the proximal tibia, with cortical sharpness, with no ruptures.

Non-ossifying fibromas are quite common fibrous lesions of bone that occur predominantly in the young and tend to resolve over time. They are radiolucent lesions with sclerotic borders, typically occurring at eccentric locations. These fibroids are located eccentrically at the metaphysis, appearing as bony lesions in the cortical bone, and often have a multilocular appearance (Figure 6). Another type of benign bone lesion is fibrous cortical defect, which is histologically identical to non-ossifying fibroma but usually smaller than 3.0 cm, presenting as bony lesions in the cortical bone that become sclerotic as they progress toward healing^(^1^,^^2^^,^^6^^)^.

### Simple bone cyst

**Figure 7:**
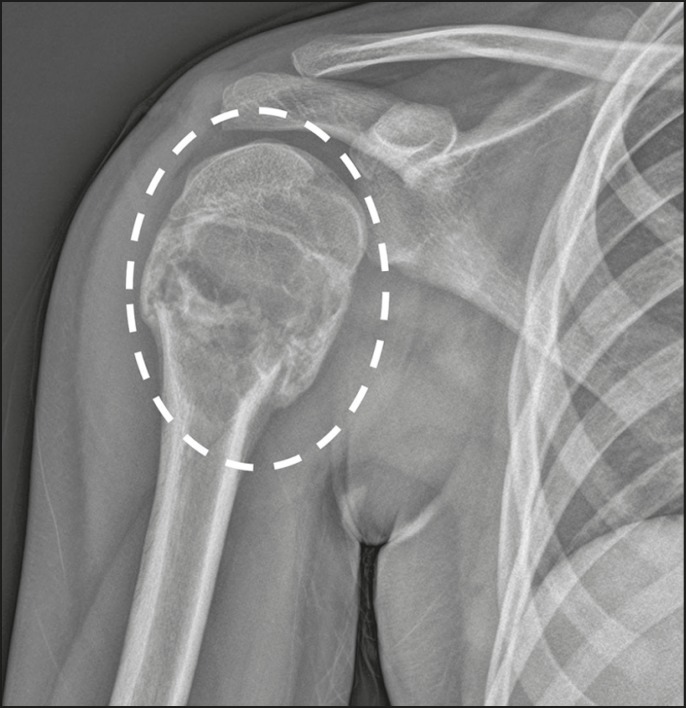
A 12-year-old male with a two-day history of right shoulder pain and no history of trauma. An X-ray of the shoulder shows a lytic lesion without aggressive features in the proximal metadiaphyseal region of the humerus, presenting a transverse fracture line with mild medial impaction and signs of developing consolidation, in addition to a dislodged bone fragment within the lesion. The radiographic appearance suggests a simple bone cyst (dashed ellipse).

Simple bone cysts are alterations that typically occur prior to the third decade of life and are preferentially intramedullary, typically occurring in the proximal femur or proximal humerus. They are lytic lesions that are well delimited by sclerotic margins, centrally located, and typically without periosteal reaction except when associated with fractures (Figure 7). In cases of associated fractures, a typical finding is a signal from a dislodged fragment^(^1^,^^2^^,^^6^^)^.

### Aneurysmal bone cyst

**Figure 8:**
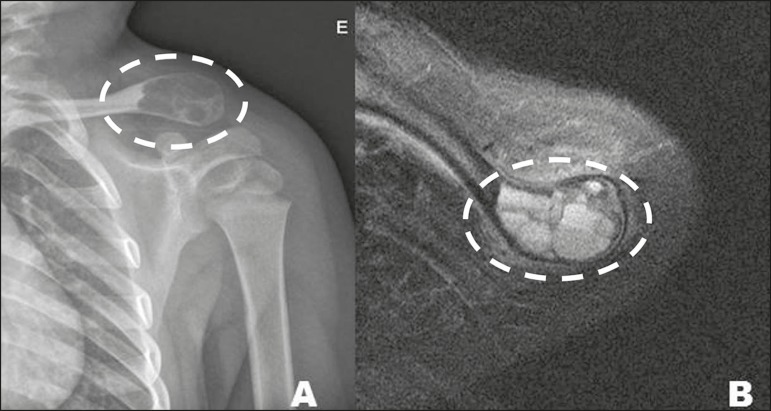
A 2-year-old male examined five days after a fall. An MRI scan shows a multilocular cystic expansile osteolytic lesion (dashed ellipse), observed as an incidental finding, in the lateral third of the clavicle, resulting in cortical thinning and narrowing, with a fluidfluid level. The lesion had no softtissue or solid components.

Aneurysmal bone cysts are typically found in young adults up to 30 years of age and can occur in association with other bone lesions, in which case they are said to be secondary. The typical presentation is that of an eccentric, expansile, multicystic lesion, with or without an adjacent periosteal reaction. MRI shows a lesion with lobulated contours containing fine septations, representing multicystic cavities. It is common to see hypointense margins and fluid-fluid levels within the cysts (Figure 8).Despite being classified as benign cystic lesions, up to one third of aneurysmal bone cysts are secondary to underlying diseases and thus require interventions, such as curettage and bone grafting^(^1^,^^2^^,^^6^^)^.

### Bone infarction

**Figure 9:**
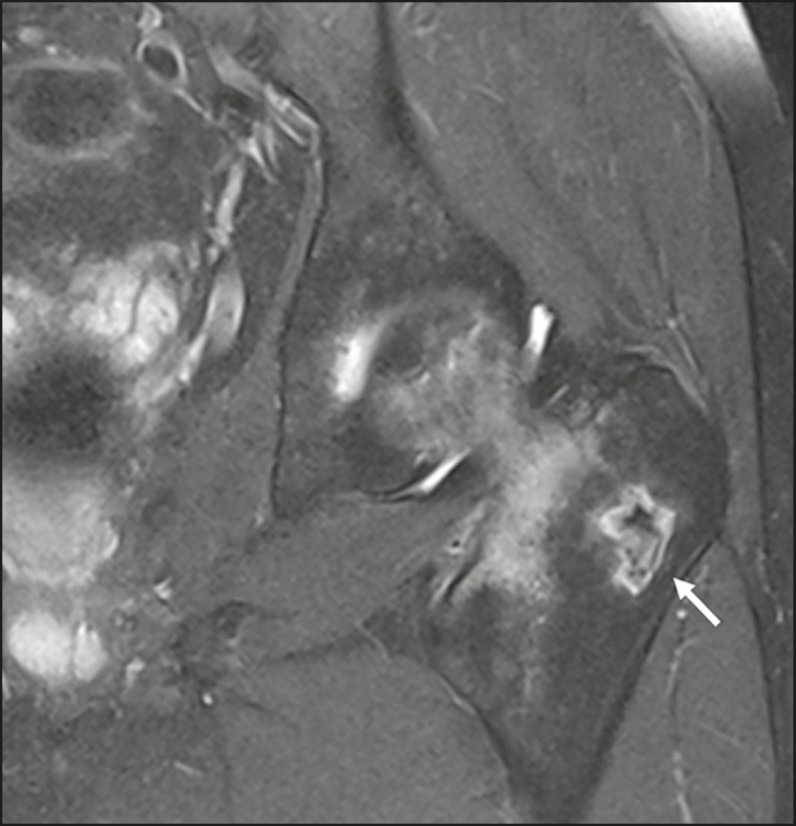
A 14-year-old male with a six-month history of meningitis, which was still under treatment, and a more recent history of pain in the left hip. MRI shows osteonecrosis of the left femoral head, accompanied by marked edema extending to the proximal metaphysis, without joint collapse or fracture of the loading area. Note the small focus of bone infarction, with a geographic pattern (arrow), in the major trochanter of the left femur.

Early findings on conventional X-rays can range from lytic lesions to foci of bone sclerosis. With maturation, there is delimitation of the lesion, which becomes more sclerotic and begins to present serpiginous or geographic borders and a radiolucent periphery. On MRI, findings in the most acute phase include circumscribed lesions with bone edema, showing serpiginous or geographic borders with low signal intensity. Contrast use can reveal margin enhancement and a center with low signal intensity (Figure 9), following the pathophysiology of the disease, in which the central region represents the infarct area, where there is inadequate blood supply^(^2^,^^6^^)^.

### Synovial cysts

Synovial cysts present as radiolucent foci in the anterior superior portion of the femoral neck (Figure 10). They are thought to be a consequence of herniation of the synovium into cortical defects and might be related to femoroacetabular impingement^(^1^,^^2^^,^^6^^)^.

**Figure 10:**
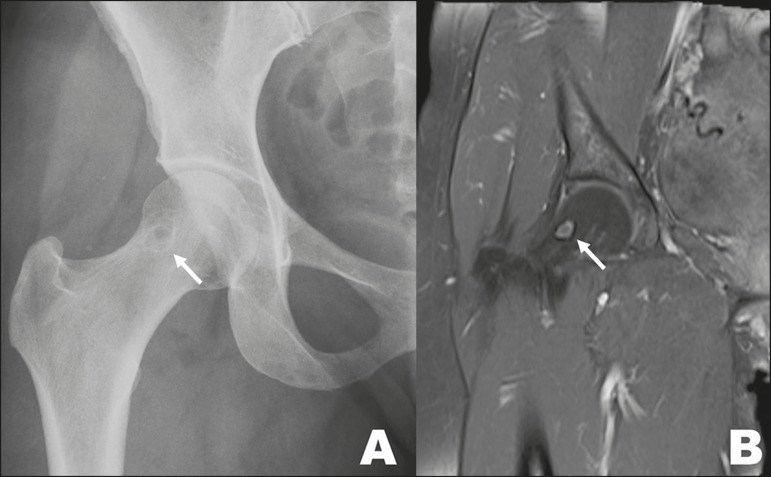
10. A 46-year-old female with a history of L5-S1 disc herniation, with a synovial cyst (arrow), identified as an incidental finding on X-ray (**A**) and MRI (**B**), in the right femoral neck.

### Melorheostosis

**Figure 11:**
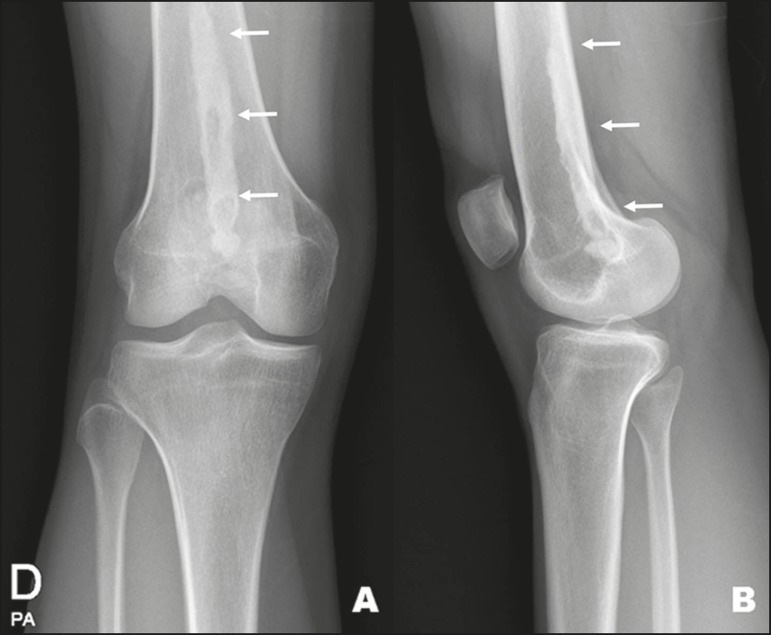
A 26-year-old female with a two-week history of left knee pain and no history of trauma. X-ray of the lower limbs, obtained to investigate asymmetry, revealed an additional finding of textural alteration with linear bone sclerosis (arrows) in the distal and posterior contour of the right femur, characteristic of melorheostosis.

Melorheostosis is an uncommon form of mesenchymal dysplasia. It manifests as areas of sclerotic bone with the appearance of melted candle wax^(^1^,^^2^^,^^6^^)^, as depicted in Figure 11.

### Vertebral hemangiomas

Vertebral hemangiomas are the most common benign vertebral neoplasms. On X-rays, they appear as thickened trabeculae with a vertical orientation. In an axial CT slice, this pattern of thickened trabeculae takes on a "fluted" appearance^(^1^,^^2^^,^^6^^)^.

### Discogenic vertebral sclerosis

Discogenic vertebral sclerosis typically presents as sclerotic focal lesions adjacent to the vertebral plateau, with narrowing of the underlying disc space, occasionally accompanied by osteophyte formation^(^1^,^^2^^,^^6^^)^

## POST-TRAUMATIC LESIONS

### Myositis ossificans

**Figure 12:**
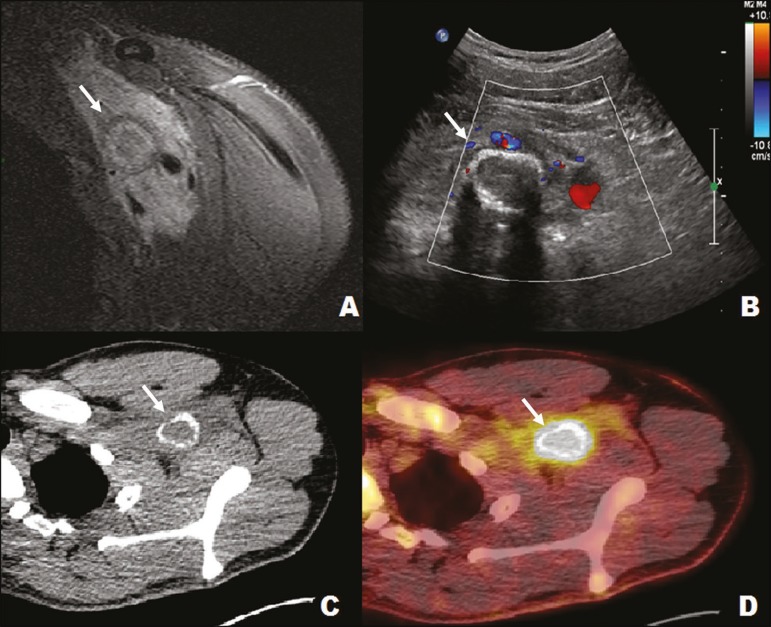
A 22-year-old male with left shoulder pain after trauma. MRI of the left axillary region (**A**) showing a tumor (arrow) involving neural vessels and bundles. Ultrasound (**B**) showing an infiltrative muscle lesion (arrow), with peripheral calcification in the pectoral/left axillary region and no detectable vascularization on the Doppler flow study. Positron emission tomography (**C**) and CT (**D**) showing an infiltrative lesion (arrow) in the left retrosternal/axillary region, with a marked increase in glycolytic activity. The imaging aspect, together with the clinical history, was definitive of myositis ossificans.

Myositis ossificans is a benign condition in which bone tissue forms within muscle or other soft tissue after an injury. It most often occurs within the large muscle groups of the extremities and typically affects young adults. In the first two weeks, it presents as a soft-tissue mass and edema. Bone deposition in the lesion begins in the third to fourth week, delimiting a border of peripheral bone with a central lucent area. Up to the sixth month, the most peripheral bone tends to mature, while the central zone presents an immature matrix, the so-called zonal phenomenon, which can be well characterized on imaging. Thereafter, the lesion tends to involute^(^5^,^^7^^)^. An MRI scan shows the various phases of the lesion (Figure 12). Conventional X-ray and CT are both effective methods of characterizing bone formation and the zone phenomena. The radiologist plays a central role in the evaluation of myositis ossificans, given that, in a biopsy performed in the early stages, it will be indistinguishable from a sarcoma; the responsibility for making an accurate diagnosis therefore falls on the radiologist^(^5^,^^7^^-^9^)^.

## NORMAL ANATOMICAL VARIANTS

### Humeral pseudocyst

**Figure 13:**
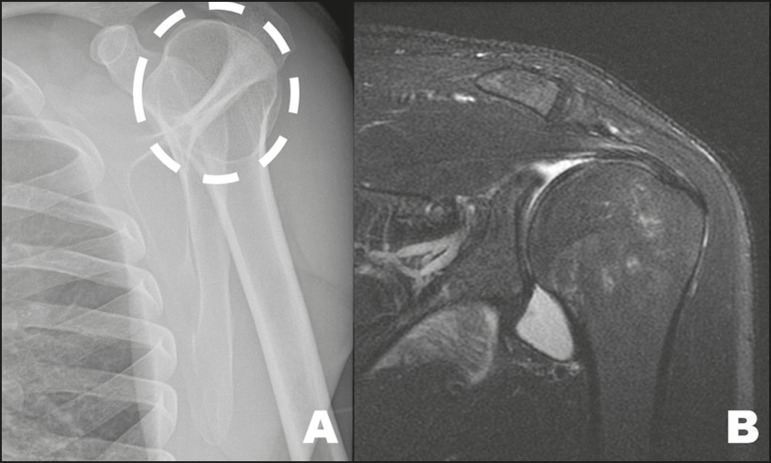
A 27-year-old male examined one hour after a bicycle accident. Note the image suggestive of a humeral cyst (dashed circle) on the X-ray (**A**), an incidental finding that was not confirmed in complementary views or in MRI study (**B**), indicating that it was a humeral pseudocyst.

A humeral pseudocyst is a radiolucent area located in the major tuberosity of the humerus, which can be seen on conventional X-rays (Figure 13). Although it is considered a normal anatomical variant, it can mimic a lytic lesion^(^1^,^^2^^,^^6^^)^.

## CONCLUSION

It is essential that the radiologist knows the differential diagnoses of lesions that mimic bone tumors with aggressive malignant potential, in order to avoid performing unnecessary invasive procedures and placing a heavy psychological load on the patients.

We hope that this small, illustrative review will help our colleagues increase their accuracy in diagnosing these conditions, in which the radiologist plays a fundamental role in avoiding the serious diagnostic errors caused by unnecessary biopsies, as well as the catastrophic consequences of such errors.
